# Characteristics of Rheoencephalography and some Associated Factors on Menopausal Women

**DOI:** 10.2478/joeb-2022-0012

**Published:** 2022-12-18

**Authors:** Tin Hoang Nguyen, Kien Trung Nguyen, Long Duc Tran, An Thi Thuy Le, Thu Minh Phung, Truc Thi Ngoc Banh, Trang Thi Vo, Michael Bodo

**Affiliations:** 1 Faculty of Medicine, Can Tho University of Medicine and Pharmacy, Can Tho, Vietnam; 2 Department of Functional Diagnostics, Can Tho University of Medicine and Pharmacy Hospital, Can Tho, Vietnam; 3 Department of Neurocritical Care, Ochsner Medical Center, New Orleans, LA, USA; 4 Uniformed Services University of the Health Sciences, Bethesda, MD, USA

**Keywords:** rheoencephalography, menopause, central obesity, women, Vietnam

## Abstract

The significant drop in estrogen levels during menopause increases the cardiovascular risks, one of which is cerebrovascular atherosclerosis. Research on rheoencephalography (REG) parameters for the early diagnosis of cerebrovascular atherosclerotic lesions is of great interest to scientists because of its ease of implementation, low cost, and non-invasiveness. The objectives of study are to evaluate the vascular tone, cerebral circulation flow in each hemisphere of the brain of menopausal women, and some associated factors through waveform characteristics and parameters in REG. A controlled cross-sectional descriptive study was conducted on a group of patients including 80 menopausal women and a control group of 46 menstruating women. All patients were measured REG in the frontal-occipital leads by VasoScreen 5000 impedance REG meter. In menopausal women, the percentage of sharp waves, the percentage of clear side waves, and the average REG were all lower than in the control group (p<0.01). The mean conduction time and mean slope ratio was lower than the control group (p<0.001). The mean peak time was higher than the control group (p<0.01). The mean elasticity index (alpha/T) was higher than the control group (p<0.001). Menopausal women have increased vascular tone, the highest in the group of women 50-60 years old, menopause <5 years, having a habit of eating red meat; and decreased blood flow intensity, the highest in the group of women <50 years old. However, the difference was statistically significant only in the left hemisphere (p<0.05). Vascular hypertonia in menopausal women with central obesity was higher than in the non-obese group in both hemispheres (p<0.05). In conclusion, menopausal women had atherosclerosis in both hemispheres of the brain, which was clearly shown in the rate of increased vascular tone. Central obesity may increase the risk of vascular hypertonia 3.75 times in the right and 5.44 times in the left hemisphere.

## Introduction

In recent years, human life expectancy is gradually increasing, leading to an increase in the proportion of women going through menopause. In 1990, there were about 467 million postmenopausal women worldwide, but it is expected that by 2030, the number of menopausal and postmenopausal women will increase to 1.2 billion with 47 million new arrivals each year [1]. In developed countries, the average woman is expected to spend about one-third of her life postmenopausal [2]. Therefore, health care for menopausal women has become a matter of great concern to the community.

Menopause is marked by a drastic change in hormonal balance, with an initial decline in estrogen leading to high levels of Follicle Stimulating Hormone (FSH) and Luteinizing Hormone (LH), and eventually with a decrease in progesterone levels and permanent amenorrhea [1]. The average age of menopause in women is 49 years [3]. During perimenopause, when estrogen levels drop, usually from the mid-40s onwards, the protective effect is lost, and changes occur that gradually lead to an increased risk of heart disease in subsequent years [4]. Under the age of 45, men are twice as likely to develop cardiovascular disease as women. This gap decreases with age until after women reach menopause. The risk of cardiovascular disease increases in women with each year of menopause and eventually surpasses men [5-6]. Entering menopause, women have estrogen deficiency (the main cause) and have a marked increase in the percentage of android fat, a diminishment in the gynoid fat ratio. This is considered one of the risk factors for atherosclerosis, especially in organs such as the brain, heart, and kidneys [7, 8, 9].

**Fig.1:**
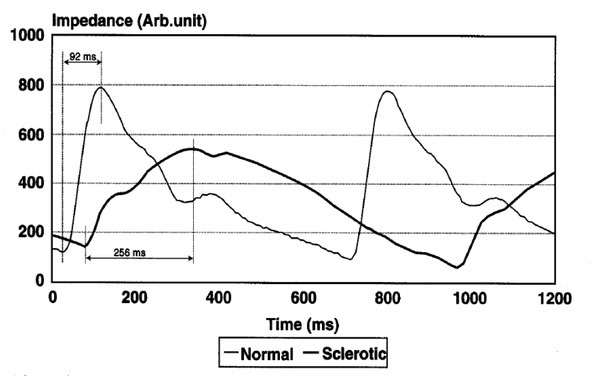
Typical REG curve of a healthy (normal) and a sclerotic person. Note the difference in the peak time of REG curves for normal patients (92 milliseconds) and sclerotic patients (256 milliseconds). This difference has pathological meaning, independent of heart rate [18].

Doppler ultrasound of cerebral blood vessels is currently used to assess cerebral vascular injury because it is non-invasive and allows for an accurate diagnosis of both morphology and hemodynamics. However, pulse Doppler ultrasound necessitates the sonographer's experience, is relatively costly, and is only available in a few specialized medical facilities. Furthermore, the implementation process is time-consuming and unsuitable for screening programs. As a result, REG is an option to consider. REG is a valuable method for assessing cerebrovascular status and cerebral circulation. REG can detect cerebrovascular atherosclerosis at a more presymptomatic stage than Doppler ultrasound [4], [10]. This is also a non-invasive technique, which is easy to perform and can be used for continuous measurement in the diagnosis of cerebral atherosclerosis [11-12]. Although REG has less clinical value in the diagnosis of cerebrovascular disease, it may provide information on changes in cerebral circulation which occurs with aging or diffuse cerebrovascular disease [13]. Especially, there is typical differences between REG waves of a normal and a sclerotic person (Fig.1) [18]. However, surveys for early detection and assessment of cerebral atherosclerotic lesions through assessment of cerebral circulation in Vietnam were still of little interest. Currently, Can Tho University of Medicine and Pharmacy Hospital is one of the pioneers in this field, especially in the Mekong Delta and the South of Vietnam in general [14-15].

**Fig.2:**
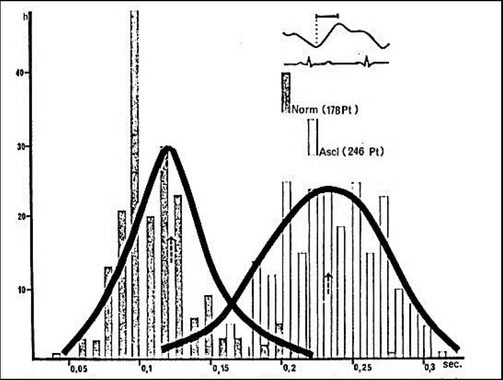
Diagrammatic presentation of compiled data on time (in seconds) between the onset of a phase and highest (first) peak, averaged from 100 phases of each patient (Pt) of 178 normal (Norm) subjects and 246 patients with proven cerebral arteriosclerosis (Ascl). All of which had been submitted to carotid arteriography. Unit of the Y-axis is the incidence or case number (frequency) [38].

Although there have been several research projects in REG, these are mainly about cerebrovascular accidents or chronic diseases such as primary hypertension, type 2 diabetes, and metabolic syndrome. Therefore, our research group conducted this study with two objectives: *Studying vascular tone, and cerebral circulation flow in each hemisphere of the brain in postmenopausal female patients and investigating some factors related to increased vascular tone and decreased REG intensity with cerebral vascular disease in menopausal female*.

## Materials and methods

### Research object

The study was conducted on menopausal female patients who came to the Department of Functional Exploration, Can Tho University of Medicine and Pharmacy Hospital from January 1^st^, 2020, to December 12^th^, 2020. Besides, a control group of female patients who were still menstruating going to a general health check or be prescribed by a doctor to measure REG. Subjects were invited to participate in the study by means of convenient sampling during the study period until the number of subjects was sufficient.

In the group of patients, women were selected with natural menopause, amenorrhea for more than 2 years, and no return of menstruation. The age of menopause was 40-55 years old. Women with a history of traumatic brain injury or surgery, history of cerebrovascular disease such as cerebral infarction, cerebral hemorrhage, non-atherosclerotic carotid artery stenosis (aneurysm, arteritis, arterial dissection, vasospasm, tissue damage after radiation therapy) or taking any medication that can cause vasodilation or vasoconstriction were excluded. We did not invite women who were suffering from premenopausal disorders, in the acute phase of a medical condition, having serious comorbidities, having a mental illness, or not cooperating well.

In the control group, we selected women who were on their period because estrogen levels were the lowest. The age range spread from 16 to 40. In addition to the exclusion criteria of the group of diseases, we did not include in the study cases of menorrhagia, heavy bleeding due to diseases affecting female sex hormones such as polycystic ovaries, obesity, diabetes, thyroid tumor, etc., or other diseases affecting menstruation such as uterine fibroids, uterine cancer, bleeding disorders, etc.

### Methods

A controlled cross-sectional descriptive study was conducted on 126 patients, in which the group consisted of 80 postmenopausal women and the control group had 46 menstruating women. We compared the REG characteristics in each hemisphere of the female patients with menopause and menstruation, including the following parameters:

Characteristics of REG waveforms include peak wave (sharp, obtuse, domed) and subwave (clear, blurred, absent). Normally the curve has sharp peaks and clear subwaves.

Characteristics of parameters to evaluate cerebral vascular tone include 4 values. Peak time is the time from the first starting point to the vertex of a REG wave. Peak width is the time between two points on either side of the vertex of a REG wave. Conduction time is the time from the beginning of the QRS complex of the electrocardiogram to the starting point of a REG wave. Elasticity index (alpha/T) is the percentage between the ascending branch time (α) and the duration of a REG wave cycle (T).

Characteristics of parameters assessing blood flow intensity include 3 values. Slope ratio is the proportion of the maximum slope of the ascending branch to the preset background impedance of the device. Alternating blood flow (ABF) is the percentage of blood volume in each hemisphere of the brain in 1 minute. Impedance ratio is the rate of the peripheral impedance to the background impedance.

The best illustration of how REG development is a multidisciplinary subject can be followed by the used data processing. In 1970-es the REG filtering was realized by using analogue filter. Additional data processing modules were also built based on analogue circuitry [35]. Evaluating cerebrovascular atherosclerotic status based on criteria of increased vascular tone and decreased blood flow intensity in REG according to Osadchikh and Ronkin, 1976 [28].

In addition, this article investigated some factors related to REG abnormalities including age, age of menopause, years of menopause, eating habits, history of hypertension, type 2 diabetes, dyslipidemia, Body Mass Index (BMI is a patient's weight in kilograms divided by the square of height in meters), and central obesity diagnosed according to the World Health Organization (WHO) classification for Asians [16-17]. Measurement of height, weight, waist circumference, and hip circumference were conducted according to the recommendations of WHO (2004) [33].

**Fig.3:**
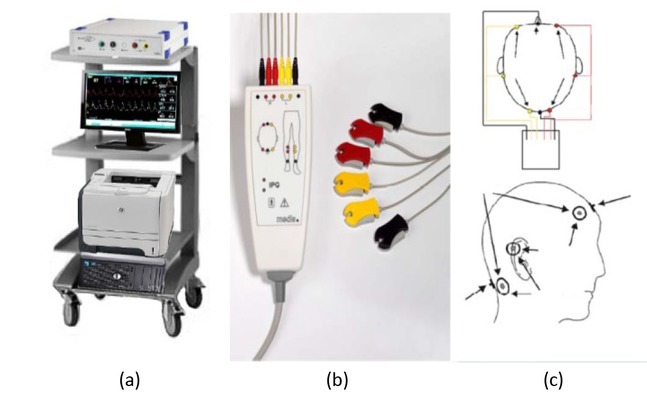
(a) REG monitoring device, (b) system of electrodes, (c) electrode attachment sites.

VasoScreen 5000 REG meter (Medis GmbH, Ilmenau, Germany) was used in our research. The device belongs to the REG II model (2^nd^ generation REG recorder). It was a dual-channel meter (left, right) with one ECG channel (Fig.3a). Measuring method based on 6 electrodes including 2 emitters and 4 receivers (Fig.3b). Rheography impedance had measuring current (1.5mA_rms_ ±1%, 85 kHz), basic impedance (0-200 Ohm ±1%, 0-1.5 Hz), impedance change (±6.25 Ohm ±2%, 0-1.5 Hz), pulse wave (±500 mOhm ±2%, 0.2-120 Hz), noise (<1 mOhm), and application (head, legs, arms). An electrocardiogram channel had input voltage (maximum ±10 mV AC 0,2-120 Hz), CMMR (>90 dB), noise voltage (<10 μV), and test signal (1 mV). The main supply was set with 115-230 V ±10%, 50-60 Hz, and 60 VA. The machine used round aluminum electrodes with a diameter of 2 cm (8 pieces). Convention was that red electrode wire was right, yellow was left (Fig.3c). REG pulse wave was analyzed by CardioVascular Lab software. Certainly, data in tables 2 and 3 were created by CardioVascular Lab software.

The patient was explained and guided on the steps to measure REG so that the patient understood and cooperated well in the process. Before taking measurements, each patient was taken anthropometric measurements (weight, height).

There were some steps to measure REG. First of all, the patient was measured in a comfortable sitting position, with his back and head straight on a recliner, eyes closed, and breathing evenly during the measurement. We used a rubber band to attach the electrode. Then we tied the rubber band over the landmarks: the middle of the forehead in front, the occipital bone at the back, passing through the mastoid area behind the ear. We measured blood flow parameters simultaneously on both

hemispheres in the frontal-occipital leads and saved the blood parameters in this lead with the name Head-partial 3 (beginning of part 3). The blood circulation indicators after recording would be printed on paper and saved on a computer for analysis. The length of processed REG pulse waves was about 15 to 20 minutes. There were 2 pulse waves of the frontal-occipital leads including left and right side averaged.

All data were processed using SPSS 20.0 software (IBM, Armonk, NY, USA) according to the medical statistics method. To check the normal distribution of data, we used Kolmogorov-Smirnov test. To compare the mean difference between the two groups, Student T-test was applied with statistical significance determined when p≤0.05. To compare the proportional difference between 2 or more groups, we used the Chi-squared test (χ^2^) with statistical significance determined when p≤0.05. Odds ratio (OR) with 95% confidence interval (CI) was used when testing the relationship between 2 qualitative variables, applied to a 2x2 table.

### Informed consent

Informed consent has been obtained from all individuals included in this study.

### Ethical approval

The research related to human use has been complied with all relevant national regulations and institutional policies and in accordance with the tenets of the Helsinki Declaration and has been approved by the author’s institutional review board or equivalent committee. The study was carried out after acceptance by the Ethics Committee in Biological Research at Can Tho University of Medicine and Pharmacy on May 27^th^, 2020.

## Results

### Characteristics of the research object

According to Table 1., the average age of menopausal women was 59.60 ± 8.79 years old, in which the age group >60 accounts for the highest percentage. Moreover, most patients had menopause age 45 - 50 years old and mostly menopause <5 years. Besides, most menopausal women were obese (30.2%), and the central obesity rate was up to 87.5%.

**Table 1:** Characteristics of menopausal women

Characteristics Number	(n = 80)	Percentage (%)
**Age (years)** Mean ± SD: 59.60 ± 8.79 (Min: 43 - Max: 85)		
<50	9	11.25
50 – 60	34	42.5
>60	37	46.25
**Age of menopause (years)** Mean ± SD: 49.55 ± 3.66 (Min: 41 - Max: 57)		
<45	6	7.5
45-50	44	55
>50	30	37.5
**Time of menopause (years)** Mean ± SD: 10.1 ± 8.1 (Min: 1 - Max: 34)		
<5	30	37.5
5 – 10	18	22.5
10 – 20	21	26.25
>20	11	13.75
**BMI (kg/m^2^)** Mean ± SD: 24.44 ± 3.15 (Min: 18 - Max: 33)		
<18.5 (skinny)	1	0.8
18.5 – 22.9 (normal)	21	16.7
23 – 24.9 (overweight)	20	15.9
≥25 (obese)	38	30.2
Central obese	70	87.5

### Characteristics of REG in each hemisphere of the brain of menopausal women

REG in the menopausal group mainly had obtuse waveforms. At the same time, the sharp wave ratio was lower, and the dome wave ratio was higher than that of the control group in both hemispheres (p<0.01). In addition, subwaves in the menopausal group were often blurred or absent, while these in the control group were mainly clear or blurred. Moreover, the incidence of obvious subwaves in the menopausal group was lower than that in the control group in both hemispheres, with p<0.001. In addition, the menopausal group had a higher rate of atherosclerosis in both hemispheres of the brain, including increased vascular tone and decreased blood flow intensity, at a higher rate than the menstruating group. The difference was clearly seen in the rate of increased vascular tone, with p<0.01 (Table 2).

**Table 2:** Characteristics of REG in each hemisphere of the brain in the group of menopausal and menstruating women

Characteristics			Right hemisphere			Left hemisphere		
			Menopause (n=80)	Menstruation (n=46)	p	Menopause (n=80)	Menstruation (n=46)	p
Waveshape	Peak wave	Sharp	21 (26.2%)	23 (50.0%)	0.001*	23 (28.8%)	24 (52.2%)	0.004*
		Obtuse	39 (48.8%)	22 (47.8%)		42 (52.5%)	21 (45.7%)	
		Domed	20 (25.0%)	1 (2.2%)		15 (18.7%)	1 (2.1%)	
	Subwave	Clear	11 (13.8%)	20 (43.5%)	<0.001*	13 (16.2%)	21 (45.6%)	<0.001*
		Blurred	45 (56.2%)	25 (54.3%)		47 (58.8%)	24 (52.2%)	
		Absent	24 (30.0%)	1 (2.2%)		20 (25.0%)	1 (2.2%)	
REG Parameters	Peak time (ms)		212.19 ±29.48	192.11 ±37.10	0.001**	211.64 ±30.33	186.43 ±41.26	<0.001**
	Peak width (ms)		74.89 ±10.18	89.89 ±20.33	<0.001**	75.31 ±9.75	88.54 ±15.11	<0.001**
	Conduction time (ms)		112.48 ±14.89	124.65 ±18.60	<0.001**	111.43 ±15.16	123.89 ±19.09	<0.001**
	Elasticity Index alpha/T (%)		28.36 ±2.28	25.70 ±3.00	<0.001**	28.26 ±2.28	25.51 ±3.94	<0.001**
	Slope ratio (‰/second)		8.61 ±2.46	±3.38	11.77<0.001**	9.33 ±2.70	12.53 ±3.22	<0.001**
	ABF (%/minute)		19.47 ±5.08	23.17 ±7.32	0.001**	20.85 ±5.02	24.89 ±8.22	0.002**
	Impedance ratio (‰)		0.91 ±0.24	0.96 ±0.24	0.309**	0.98 ±0.25	1.01 ±0.23	0.449**
Disorders	Increased vascular resistance (%)		67.5	30.4	<0.001*	65.0	28.3	<0.001*
	Decreased intensity of blood flow (%)		16.2	8.7	>0.05*	13.8	4.3	>0.05*

*: Comparison between menopause group and menstruation group (Chi-Square Tests). **: Comparison between menopause group and menstruation group (t-test for Equality Means).

### Some factors related to atherosclerosis

In the left hemisphere, the rate of hypertonia was highest in the age group 50-60 years old and the rate of decrease in blood flow was highest in the age group <50 years old. At the same time, the rate of increased vascular tone in the group of menopause <5 years accounted for the highest rate (83.3%), with a p<0.05. Additionally, menopausal women with a habit of eating red meat had the highest rates of vascular hypertonia and decreased blood flow, a statistically significant difference was only found when assessing the rate of increased vascular tone in the left hemisphere. Besides, the rate of vascular hypertonia in the group of women with central obesity was statistically significantly higher than in the group without central obesity when evaluated in both hemispheres (p<0.05). Especially, central obesity was associated with a 3.75 times increased risk of right cerebral vascular hypertonia and a 5.44 times increased risk of left cerebral vascular hypertonia (Table 3).

**Table 3:** Some factors related to abnormalities in REG

Characteristics		Right hemisphere				Left hemisphere			
		Increased vascular resistance	p	Decreased intensity of blood flow	p	Increased vascular resistance	p	Decreased intensity of blood flow	p
Age (years)	<50	77.8	0.058	33.3	0.122	77.8	0.004	44.4	0.016
	50-60	79.4		20.6		82.4		11.8	
	>60	54.1		8.1		45.9		8.1	
Time of menopause (years)	<5	83.3	0.131	20.0	0.463	83.3	0.020	16.7	0.463
	5-10	61.1		16.7		66.7		11.1	
	10-20	57.1		19.0		52.4		19.0	
	>20	54.5		0.0		36.4		0.0	
Protein eating habit	Vegan	75.0	0.206	0.0	0.057	50.0	0.046	0.0	0.084
	Red meat	81.8		31.8		86.4		27.3	
	White meat	61.1		11.1		57.4		9.3	
History of hypertension (JNC 7)	Grade II	73.3	0.802	16.7	0.997	70.0	0.521	10.0	0.578
	Grade I	64.3		14.3		57.1		7.1	
	Pre-hypertension	66.7		16.7		70.8		20.8	
	Normal	58.3		16.7		50.0		16.7	
History of diabetes	Type 2 diabetes	64.3	0.777	7.1	0.309	50	0.195	7.1	0.429
	Normal	68.2		18.2		68.2		15.2	
History of dyslipidemia	Dyslipidemia	73.3	0.388	16.7	0.938	66.7	0.809	13.3	0.933
	Normal	64.0		16.0		64.0		14.0	
BMI	Obese	65.8	0.335	13.2	0.144	65.8	0.378	10.5	0.238
	Overweight	80.0		30.0		75.0		25.0	
	Skinny or normal	59.1		9.1		54.5		9.1	
Central obesity	Likely to have	71.4	0.047*	17.1	0.567	70.0	0.013**	85.7	0.713
	Not to have	40.0		10.0		30.0		90.0	

(*: OR = 3.75, CI 95% = 0.96 - 14.72; **: OR = 5.44, CI 95% = 1.28 - 23.12)

## Discussion

### Characteristics of the research object

Our study was conducted on 80 menopausal women between the ages of 43 and 85, the mean age was 59.60 ± 8.89 years. This result is like the study of Nguyen Dinh Phuong Thao on 1110 menopausal women with an average age of 56.11 ± 4.33 years old, the lowest age 42, the oldest age 65, and the women aged from 42 to 60 making up the majority with 83.4% [9]. The selection of the appropriate control group to be like the patient group poses many challenges for the research team. To avoid perimenopause, the subjects were targeted as women with amenorrhea for more than 2 years and the control group were young women <40 years of age. In addition, age is a factor associated with menopause in women, so it is difficult to avoid the age difference between menopause and menstruating groups. This is a limitation of the study, leading to a significant age difference between the 2 groups. High estrogen levels during the sexually active phase have a protective role in the early manifestations of cardiovascular disease by maintaining high density lipoprotein cholesterol (HDL-c) and low-density lipoprotein cholesterol (LDL-c) levels. The absence of endogenous estrogen during menopause causes this ratio to shift in the opposite direction, allowing atherosclerotic lesions to form [6]. In addition, the selection of a control group in menstruating period with the lowest desired estradiol concentration, <50 pg/ml [20]. However, to evaluate the similarity between the two study groups, it is necessary to quantify sex hormone levels in the blood, which in this study we are not qualified to conduct.

The mean BMI of the menopausal women was 24.44 ± 3.15 kg/m^2^ in the overweight group according to the WHO classification for adults in Asia [16]. This index is like the results of other studies of 25.1 ± 3.6 kg/m^2^ [21] and 24.2 ± 3.2 kg/m^2^ [22], respectively. The central obesity rate was the majority in the group of menopausal women, specifically in Yong Sang Son's study 54.61%, and in our study, up to 87.5% [22].

### Characteristics of REG in menopausal women

REG in the group of menopausal women mainly had obtuse waveforms (48.8% on the right and 52.5% on the left). At the same time, the spike-wave rate was lower, and the dome wave rate was higher than that of young women in both hemispheres, the difference was statistically significant with p<0.01. Besides, the side waves were often faint or absent, while in the group of young women mainly clear or fuzzy subwaves, the incidence of obvious subwaves was lower in the group of menopausal women than in the control group in both hemispheres with p<0.001. The results of our study have similarities with some studies in REG on other subjects. In the group of patients with MetS (metabolic syndrome), the study also showed that cerebral hemorrhage waveform disorder appeared. Mainly obtuse and domed waves were 96.9% and subwaves blurred or unclear were 75.0% on the right side and 78.1% on the left side [14]. In hypertensive patients, the highest percentage of obtuse waves was 72.0% [23]. Studying in patients with digital cerebral angiography with background erasure with stenosis showed obtuse and domed waves accounting for 94.1% (extracranial stenosis group had the rate of obtuse and domed waves up to 100%), opacity or loss accounts for 100% (both extracranial and intracranial) [24]. The loss of the side wave crest occurs in nearly all cases of atherosclerosis [25]. Although the REG waveform does not often accurately reflect the stages of cerebral atherosclerosis, when it is changed, it is often proportional to the degree of atherosclerosis [26]. In the stages of atherosclerotic disease, the waveform is initially rounded or slightly flat (obtuse), the subwaves appear blurred, later the wave peaks are dome, plateau, and the subwaves are very faint or do not appear. This represents increased cerebral vascular tone, i.e., decreased elasticity of the vascular wall [27].

The menopausal women's REG had a higher peak time parameter than the menstruating group in both hemispheres, with p≤0.001. Meanwhile, conduction time parameters in both hemispheres were statistically significantly lower than in the menstruating group, with p<0.001. Furthermore, the elasticity index (alpha/T) was higher in both hemispheres than in the menstruating group (p<0.001). Although the peak width parameter value was lower than that of the menstrual group and within the normal range, it did not meet Osadchikh and Ronkin's (1976) diagnostic criteria for increased vascular tone, so it had no effect on the overall results of our study. The study showed that in the stage of obvious atherosclerosis, the peak time lasted from 200-230 ms or more. As the degree of atherosclerosis increases, vascular elasticity decreases, which leads to increased cerebral vascular tone manifesting an increase in peak time [27]. Similarly, a research showed that 246 patients with proven cerebral arteriosclerosis had a higher peak time parameter than 178 normal subjects (Fig.2) [18]. Additionally, the study by Bodo et al. in REG in 546 subjects at high risk of cerebrovascular accident also showed that increasing peak time reduces arterial elasticity. The normal standard value was 180 ms. This parameter is an early marker of cerebral atherosclerosis, and it better reflects early cerebral atherosclerosis than carotid Doppler ultrasound [10].

We discovered that menopausal women had a 67.5% (right) and 65.0% (left) increase in bilateral hemispheric schizophrenia, while the rate of decrease in blood flow intensity was 16.2% (right) and 13.8 % (left) greater than the menopausal group. When all three leads measuring REG were compared in patients with MetS, there was a higher rate of increase in vascular tone and decrease in blood flow intensity in both hemispheres than in controls [14]. The study in hypertensive patients yielded similar results, with the rate of increase in vascular tone in three leads (forehead-mastoid, mastoid-occipital, frontaloccipital) being 54%, 58%, and 58%, respectively. The rates of decrease in blood flow intensity were 50%, 48%, and 38%, respectively [23]. As for patients with type 2 diabetes, the study showed that vascular tone was higher in the group of patients than in normal people (p<0.01), clearly expressed in parameters of conduction time and elastic index (alpha/T), and at the same time, blood flow intensity in the group of patients was lower than that of normal people (p<0.01), evident in REG parameters [15]. The study in patients with cerebral artery stenosis showed that the rate of increase in stroke rate was 76.5%, the rate of decrease in stroke was 70.6%, and the rate suggestive of atherosclerosis in REG was 36.4% [24]. Thus, the great role of REG in the diagnosis of atherosclerosis in different subjects was shown by the increase in vascular tone and decrease in blood flow intensity on both hemispheres of the brain. Although the results of each study on different disease groups have differences, similarities can be seen when compared with the control group of each study. Perez and Guijarro suggested that REG waves were also influenced by the thickness of the scalp and by extracranial circulation [29, 30, 31]. Waveform differences between extra and non-extracranial components are significant and these differences could be used in a method to distinguish one from the other. However, a significant part of the REG I signal is caused by a non-extracranial source. Therefore, it should not be used as a footprint of the extracranial blood flow [32].

It is obvious that REG is a good assessment of cerebral blood flow abnormalities. For organs in the body, during recording, its resistance changes only depending on the change in the amount of blood circulating through it because other factors are constant. Therefore, monitoring the change in resistance of the organelle helps us to assess the cyclic state of that organelle. The cranium can be thought of as a model of electrical charge in which the skin, muscle, and skull bones under each recording electrode are represented by the charge cell. Thus, the change in recorded electrical resistance represents the circulation of blood through the scalp, subcutaneous tissues, and skull, but this circulation is insignificant compared to the circulation through the brain. Therefore, the resistance change recording line mainly shows the change in circulating flow through the brain. When the blood flow through the brain is less, the resistance of the brain increases and the amperage decreases [37]. Moreover, excessive inflammation and pyroptosis of vascular endothelial cells due to estrogen deficiency are among the causes of atherosclerosis in postmenopausal women. It is hypothesized that estrogen may reduce pyroptosis of vascular endothelial cells through the activation of autophagy via estrogen receptor alpha (ER-α) to ameliorate postmenopausal atherosclerosis. The results of one study demonstrated that postmenopausal women have reduced expression of autophagy and ER-α and excessive damage to the ascending aorta [36]. It is possible that hormonal conditions can have an effect on the changes of electrical properties and be reflected in REG waves. However, the measurement in our study was just one and not a longitudinal study. As a result, we can't state anything how REG pulse morphology and its change can correlate to hormonal conditions. Thanks to this, a longitudinal study is initiated to establish a correlation between arteriosclerosis, REG waveshape and hormonal condition. Currently, the theoretical basis of the measurement results and the factors affecting the REG is still controversial, which explains the variation in research results of different authors in REG in subjects of different research. In our study, the rate of vascular tone increase in menopausal women was statistically significantly higher than in the control group (p<0.001), which is a very alarming situation.

### Some factors related to atherosclerosis

Our study showed that the rate of increase in vascular tone was highest in the group of women 50-60 years old and the rate of decrease in blood flow intensity was highest in the group of women <50 years old (p<0.05). Menopausal women <5 years had the highest rate of increase in vascular tone (p<0.05). However, the association between age and years of menopause with REG was not clear because statistically significant differences appeared only in the left hemisphere. In addition, we also did not find an association between REG and age at menopause when compared between 3 groups of patients (<45 years old, 45-50 years old, >50 years old). In patients with MetS, the study results also did not show an association between age and increased vascular tone in REG [14]. Meanwhile, hypertensive patients ≥60 years old had a higher decrease in blood flow intensity through the internal carotid artery and vertebral artery - basal body compared with the group of hypertensive patients <60 years old and no difference was found in vascular tone of 2 age groups above [23]. The aging process of blood vessels always occurs in the elderly due to structural changes leading to poor response to vasoactive substances, creating conditions for atherosclerosis to occur. Therefore, age is considered a poor prognostic factor for vascular events [33].

Besides, we found that the rate of increase in vascular tone and rate of decrease in blood flow intensity in menopausal women did not have a statistically significant relationship with eating habits. Although the highest rate of increase in vascular tone occurred in the group of menopausal women with a habit of eating red meat, the difference was statistically significant only in the left hemisphere (p<0.05).

Menopausal patients with a history of chronic medical diseases such as hypertension, type 2 diabetes, and dyslipidemia had a high rate of vascular tone increasing and lower blood flow intensity in both hemispheres, with no difference when compared to menopausal women. These diseases did not affect women during menopause. It is possible that in menopausal women, even if they do not have chronic diseases associated with atherosclerosis, the decline in estrogen levels contributes to the risk of atherosclerosis that is still present. Other studies, in patients with type 2 diabetes, found no link between hypertension, dyslipidemia, and cerebral atherosclerosis as measured by REG [15]. In hypertensive patients, there was no association between dyslipidemia and increased cerebral blood flow intensity, but the dyslipidemia group had a higher decrease in cerebral blood flow intensity compared to the control group. Patients with grade III hypertension (according to The 6^th^ report of the Joint National Committee on Prevention, Detection, Evaluation, and Treatment of High Blood Pressure) had a higher reduction in cerebral blood flow intensity and increase in vascular tone compared to grade I hypertensive patients [23]. Studies on patients with MetS have not yet found an association between hypertension, hyperglycemia, and elevated LDL-c with increased vascular tone in REG [14]. In general, the studies showed that despite the differences in subjects, the chronic comorbidities had little or no relationship with the results of the measurement of REG in the patient group. This can be explained because the above studies have demonstrated that each disease is related to abnormalities in REG. With a small sample size, whether a patient has one or more additional diseases. Any other chronic condition is unlikely to lead to a clear change in REG measurement. More studies with larger sample sizes are needed to make the differences between groups with and without chronic diseases more evident.

When assessing different body types (thin - average, overweight, obese), we have not found a relationship between the body of menopausal women with changes in REG on both sides. Research on hypertensive patients has also not recorded the association between overweight - obesity with decreased cerebral vascularity and increased vascular tone in all three leads [23]. In patients with type 2 diabetes, the rate of cerebral atherosclerosis in the obese group was 78.9%, 1.2 times higher than in the non-obese group. However, no statistically significant difference was found between the two groups [15]. Thus, studies in various patient populations have revealed that obesity is not associated with abnormalities in REG. However, the link between obesity and vascular complications has been proven. Research showed that obesity not only caused insulin resistance and diabetes but also participated in lipid metabolism disorders, leading to atherosclerosis. vasculature, promote inflammation, and enhance atherogenesis independent of the effects of insulin resistance [34].

Notably, when we examined the relationship between central obesity and REG in menopausal women, we discovered that the rate of vascular tone increase in the women with central obesity was higher than in the women without central obesity when evaluated in both hemispheres of the brain (p<0.05). Central obesity was linked to a 3.75 times increase in the risk of right cerebral edema and a 5.44 times increase in the risk of left cerebral edema. Another study found that the rate of increased vascular tone in REG in centrally obese patients with metabolism disorder was higher in the frontal-mastoidal leads, comparable in the mastoid-occipital leads, and lower in the frontal-occipital leads (p>0.05) [14]. This difference may be because patients with metabolism disorder have a very high rate of central obesity. In fact, central obesity is also one of the diagnostic criteria for metabolism disorder according to the National Cholesterol Education Program (NCEP) Adult Treatment Panel - III (ATP-III) report of 2001 (updated in 2004) with a cut-off point for Asians [19]. Because of the small sample size, the difference between groups with and without central obesity may be difficult to discern. The mean thickness of the common carotid intima-media layer in MetS patients was found to have no correlation with BMI but a positive correlation with waist circumference and the waist-hip ratio (WHR) [12]. This finding is like ours. The changes in REG in menopausal women, while not related to BMI, are positively correlated with central obesity, as defined by waist circumference and WHR.
